# The Mallet

**Published:** 1860-06

**Authors:** J. Taft


### THE MALLET.

BY J. TAFT.

THE method generally adopted for consolidating gold, in filling teeth, is by pressure of the instrument with the hand alone. With perfectly formed points and skillful manipulation, by this method the work is very well accomplished, though not so perfectly as it may be done.

For consolidating the gold in filling teeth, we have for three months past, been using the mallet almost constantly. We adopted it at the suggestion of Dr. W. H. Atkinson, and from personal experience. We had always previous to this, regarded its use with distrust, entertaining the very common notion that its use would always be unpleasant to the patient, and in many cases painful, and would be very liable to produce periostitus; neither of which in our experience do we find to be true. The objections which originally existed in regard to the use of the mallet, were doubtless, real objections, that originated in the method of manipulating, the condition in which the gold was used, and especially from the kind of instruments that were employed. Gold is now used much lighter, in better condition and in smaller portions, than in the early periods of the profession, and it is thoroughly consolidated as it is introduced. Formerly the greater part of it was introduced and then consolidated, and necessarily required a great amount of force, to attain anything like a thorough condensation. Then large blunt pointed instruments were used; now, small, finely serrated points are used. Formerly, it was not unfrequent to have periostitus, as a result of the pressure in filling. Now no good operator has such results under favorable circumstances.

These circumstances and changes render the mallet now available, whereas once in the hands of almost all our operators it was not. The question naturally arises here, what are the advantages attained in the use of the mallet? Are they sufficient to compensate for the employment of an assistant? In answering one of these, both are answered.

One thing attained, is a more perfect condensation of the gold, and a more thorough welding than can be made by the gradual pressure. Every one will perceive this at the mere mention of it. The gold will be anchored in its position with much more facility than by the hand pressure. The instrument always acts under the mallet upon the designed point, does not slip from its position, and consequently there is no liability of abraiding or wounding the soft parts. It is far less fatiguing to the operator, than hand-pressure, and this is a very important item in protracted operations. It is not more unpleasant to the patient than the ordinary method of condensing. Such, however, has not been the general opinion in the profession. Almost all of our patients, after making the experiment, prefer to have the gold condensed with the mallet, rather than with the hand; this, however, may arise to some extent from a feeling of greater security, against accident, by slipping of the instrument.

We will give two extreme cases—occupying opposite extremes however—in practice with the mallet. The first was one in our own experience. It was a second, left, superior molar; pulp had been destroyed about a year; during which time there was chronic periostitus, that rendered the use of the tooth impracticable; being more or less soreness all the while. The canals of the fangs were cleaned out, and treatment by creosote and iodine employed with a view to restoration of the parts to health. The time employed by the treatment was very brief, not sufficient to mitigate the symptoms to any considerable extent; from the beginning of treatment to the end of the operation, was only about twenty-four hours. The applications were made one day and the operation performed the next. Before the operation the tooth was so sore as to be quite painful on a slight pressure; but the whole operation of filling the fangs, the pulp chambers, and building up nearly one half the posterior portion of the tooth, was accomplished at one sitting, and occupied about four hours. The gold was condensed from the points of the fangs to the completion of the filling, by the use of the mallet, and without pain from beginning to the end. My apprehensions were very serious at the first, but I was very much surprised to find that the blows from the mallet, did not produce actual pain, and quite as much surprised to find at the close of the operation that there was far less soreness than at the beginning. It continued to improve, and at the end of ten days the soreness was all gone, and the tooth quite as comfortable as though it had never been diseased, and still remains so, now about three months, and the confident expectation is, that it will continue so.

I now present a case of a very different character. About the 15th of March, I filled several teeth for Dr. W. A. P. One of the number was an incisor, having approximal decay, exhibiting medium sized cavity. There was no sensitiveness, at least from an approaching exposure of the pulp. The indications were that the tooth was perfectly healthy. The cavity was excavated and filled, the gold being consolidated with the mallet, without any thing unusual thus far. We will now introduce his own statement in regard to the result. "The next morning after the operation, while at breakfast, I found the tooth a little sore. I could not bite on it without pain; by avoiding it, as much as possible, I finished my breakfast without much inconvenience; there was tenderness at the apex of the root, which was soon followed by congestion on pressure, but as yet no visible swelling; my lip felt stiff and uncomfortable, and it was not fully under control of the muscles, yet there was no pain. Arrived at home, I took measures to subdue the irritation, but I found it did not respond to them as readily as I expected it would. I painted the gum with tincture iodine, applied the compound myrrh wash; took Iodite, Ferrii, internally, and drank a bottle of congress water every morning, before breakfast, for a week. The counter irritation and the other remedies held the inflammation somewhat in check. The tooth was but little sore, or longer than the others, and there was but little oscillation; pain slight. Tuesday there was a hard lump, as large as a small hickory nut, immediately under the left Ala Nasi, but no fluctuation. Wednesday much the same. The swelling more acceptable, having extended further down on the gum. Tooth but little sore, and no fluctuation. On Thursday the same, and swelling slightly diminished and less induration. Friday, the tension much diminished, some fluctuation, pus still deep; fair prospect of its absorption, tooth not very uncomfortable. Friday evening, pus nearer the surface, judge it to be small in quantity, absorption taking place, induration and swelling much diminished, as well as the infiltration of the lip, still difficult to talk and laugh, even if so inclined. In a fit of impatience I resolved to relieve the absorbents with the lancet, as the pus had better be removed through the mouth than through circulation. I had to cut quite deep, and brought away two drops of pus, with considerable blood. Taking care to prevent healing by first intention. The discharge continued for some time. The swelling rapidly subsided and I was myself again on Monday morning, with no visible sign of the rather disagreeable than painful swelling I had just experienced. I do not certainly know that the nerve in the tooth is dead; there are some indications that it is not, as the absence of much soreness, elongation and oscillation of the tooth are favorable symptoms. It was but little painful to pressure at any time, and it is apparently as well now as any of the adjoining teeth. The irritation unquestionably arose from the concussion from the mallet on the periosteum, at the end of the root. The external applications I so thoroughly employed, seem to have drawn the inflammation to the surface, and though never great, the soreness of the tooth was diminished at an early period. I suppose the early counter irritation and active stimulation of the absorbents, may have saved the nerve by transferring the seat of the disease to the soft parts outside of the alveolus."

The Dr. subsequently in a communication to us, gave his views more fully and at great length upon this subject, which we shall take occasion as early as opportunity may afford to transfer to the pages of the *Register*, with such remarks as we may deem proper.

This is the only case, so far as we are aware, in which any unpleasant symptoms have occurred, from the use of the mallet, in the almost constant employment of it for three months. Dr. P. had other teeth filled at the same time, by the same process, which exhibited no unpleasant symptoms. In filling, however, by any method, the small single fanged teeth require care, lest they suffer irritation from the pressure employed from condensing. I suppose the difficulty occurred in this case, from strokes of too great force being employed. But I have seen the same thing occur after condensation by hand pressure.

It is quite obvious that a tooth with two or three large fangs, and consequently a greater amount of attachment, will tolerate with impunity a greater amount of pressure or force of any kind, than one small single fang; so that as the amount of attachment is less, the amount of force or pressure for condensing fillings should be correspondingly diminished.

We shall have occasion to refer to this subject again ere long, and will reserve any farther thought till that time.

